# An oestrogen-dependent model of breast cancer created by transformation of normal human mammary epithelial cells

**DOI:** 10.1186/bcr1734

**Published:** 2007-06-15

**Authors:** Stephan Duss, Sylvie André, Anne-Laure Nicoulaz, Maryse Fiche, Hervé Bonnefoi, Cathrin Brisken, Richard D Iggo

**Affiliations:** 1 NCCR Molecular Oncology, Swiss Institute for Experimental Cancer Research (ISREC), Chemin des Boveresses 155, CH-1066 Epalinges, Switzerland; 2 St Andrews University Medical School, Bute Medical Building, St Andrews, KY16 9TS, UK; 3 Lausanne University Hospital, CHUV, CH-1011 Lausanne, Switzerland; 4 Geneva University Hospitals, 30 Boulevard de la Cluse, CH-1211 Geneva, Switzerland; 5 École Polytechnique Fédérale de Lausanne (EPFL), CH-1015 Lausanne, Switzerland

## Abstract

**Introduction:**

About 70% of breast cancers express oestrogen receptor α (ESR1/ERα) and are oestrogen-dependent for growth. In contrast with the highly proliferative nature of ERα-positive tumour cells, ERα-positive cells in normal breast tissue rarely proliferate. Because ERα expression is rapidly lost when normal human mammary epithelial cells (HMECs) are grown *in vitro*, breast cancer models derived from HMECs are ERα-negative. Currently only tumour cell lines are available to model ERα-positive disease. To create an ERα-positive breast cancer model, we have forced normal HMECs derived from reduction mammoplasty tissue to express ERα in combination with other relevant breast cancer genes.

**Methods:**

Candidate genes were selected based on breast cancer microarray data and cloned into lentiviral vectors. Primary HMECs prepared from reduction mammoplasty tissue were infected with lentiviral particles. Infected HMECs were characterised by Western blotting, immunofluorescence microscopy, microarray analysis, growth curves, karyotyping and SNP chip analysis. The tumorigenicity of the modified HMECs was tested after orthotopic injection into the inguinal mammary glands of NOD/SCID mice. Cells were marked with a fluorescent protein to allow visualisation in the fat pad. The growth of the graft was analysed by fluorescence microscopy of the mammary glands and pathological analysis of stained tissue sections. Oestrogen dependence of tumour growth was assessed by treatment with the oestrogen antagonist fulvestrant.

**Results:**

Microarray analysis of ERα-positive tumours reveals that they commonly overexpress the Polycomb-group gene *BMI1*. Lentiviral transduction with *ERα*, *BMI1*, *TERT *and *MYC *allows primary HMECs to be expanded *in vitro *in an oestrogen-dependent manner. Orthotopic xenografting of these cells into the mammary glands of NOD/SCID mice results in the formation of ERα-positive tumours that metastasise to multiple organs. The cells remain wild type for *TP53*, diploid and genetically stable. *In vivo *tumour growth and *in vitro *proliferation of cells explanted from tumours are dependent on oestrogen.

**Conclusion:**

We have created a genetically defined model of ERα-positive human breast cancer based on normal HMECs that has the potential to model human oestrogen-dependent breast cancer in a mouse and enables the study of mechanisms involved in tumorigenesis and metastasis.

## Introduction

Classic epidemiological studies on the increase in cancer incidence with age predicted that from three to six independent events would be required to convert a normal cell into a tumour cell [1]. Experimental studies have now proven that it is indeed possible to transform a cell in culture by modifying the activity of only a few critical genes. Cell lines quantitatively transformed by expressing oncogenes or inactivating tumour suppressor genes have been produced from normal fibroblasts, embryonic kidney cells and human mammary epithelial cells (HMECs) [2,3]. The genes initially chosen for these studies were those encoding simian virus 40 large T and small t antigen, activated Ras and telomerase. Subsequently it was shown that viral oncogenes can be replaced by activated *MYC *and genes targeting the retinoblastoma pathway [4]. This combination will transform HMECs, but the resulting tumours do not express oestrogen receptor α (ERα); this is an important weakness of current models, because about 70% of human breast tumours are ERα-positive.

ERα behaves quite differently in ERα-positive cell lines derived from human breast cancer and in normal human mammary epithelium *in vivo*. Oestradiol is a direct mitogen for ERα-positive cancer cell lines, but in normal human breast tissue the ERα-positive cells do not themselves divide in response to oestrogen [5]. Instead, they relay a proliferative signal to neighbouring ERα-negative cells. The barrier to proliferation of ERα-positive normal cells may explain why HMECs rapidly lose ERα expression in culture and why the transformation studies performed so far have produced ERα-negative tumour cell lines.

The target cell of oncogenic mutations in the breast is probably a stem cell or bipotent progenitor cell [6,7]. It is possible to enrich for these cells by growing primary HMECs in the non-adherent conditions previously developed for culture of neural stem cells, leading to the formation of so-called floating mammospheres [8]. The Polycomb-group gene *BMI1 *can suppress activation of the p53 and Rb pathways by silencing the expression of p14^ARF ^and p16^CDKN2A ^[9] and it has been shown to increase the rate of self-renewal of mammospheres in response to Wnt, Hedgehog and Notch signals [10]. Since Polycomb-group genes are overexpressed in breast cancer [11,12], *BMI1 *is a relevant candidate to test in HMEC transformation assays. *BMI1 *was originally identified as an oncogene that cooperates with *MYC *to induce lymphomas in mice [13], and *MYC *is commonly amplified in breast cancer, so it is reasonable to use *MYC *in a transformation protocol that includes *BMI1*. We show here that lentiviral transduction of HMECs with *ERα*, *BMI1*, *MYC *and *TERT *leads to the formation of ERα-positive tumours whose growth is dependent on oestrogen.

## Materials and methods

### Cell culture

Approval for culture of reduction mammoplasty tissue was granted by the Lausanne University Hospital ethics committee, and patients gave informed consent. The patients were healthy women with no previous history of breast cancer. All samples were confirmed by histopathological examination to be free of malignancy. Primary HMECs and human mammary fibroblasts (HMFs) were prepared by standard techniques [8,14]. HMECs were cultured in human mammosphere medium (HMM): Hepes-buffered DMEM/F12 without phenol red (Gibco, Basel, Switzerland) supplemented with 20 ng/ml EGF (Invitrogen, Basel, Switzerland), 1 × B-27 (Gibco) and 1 nM 17-β-oestradiol (Sigma, Buchs, Switzerland). B27 is a serum-free medium supplement containing antioxidants, vitamins, growth factors and hormones including progesterone [15]. HMM was used for suspension and adherent culture of HMECs; tissue culture plastic was not coated with collagen for adherent culture. HMFs and MCF7 cells were grown in DMEM containing 10% fetal calf serum. For the proliferation assays, 20,000 cells were seeded per well in six-well plates in duplicate and stained with crystal violet (Sigma) after 10 days of culture in HMM containing 1 nM 17-β-oestradiol or 1 μM fulvestrant (ICI 182,780; Torcis Pharmaceuticals, Bristol, UK). The area covered with cells was quantified with ImageJ software (NIH, Bethesda, MD, USA). For the growth curves, 20,000 cells per well were seeded in 12-well plates in duplicate in HMM containing 1 nM 17-β-oestradiol or 1 μM fulvestrant. A separate plate was used for each time point and cells were counted in a Neubauer chamber. For colony formation and SNP assays from tumours, explanted cells were grown briefly in puromycin to eliminate murine cells.

### Lentiviral vectors

The pSD-69 plasmid contains the human phosphoglycerate kinase (PGK) promoter, a Gateway *attR *cassette (Invitrogen), the mouse PGK promoter and the puromycin acetyltransferase gene cloned into pRRLhPGK.GFP.SIN18 [16]. Preliminary studies showed that the human PGK promoter is active in mammospheres and differentiated HMECs. Gateway *BMI1 *and *ESR1 *clones were obtained from Flexgene (Boston, MA, USA). The *MYC *(c-Myc) and *TERT *(hTERT) clones were provided by J Lingner and A Trumpp, respectively, and cloned into pENTR1A (Invitrogen). The *BMI1*, *ESR1*, *MYC *and *TERT *cDNAs were transferred to pSD-69 by LR recombination (Invitrogen) to give pSD-84, 82, 94 and 83. The *MYC *clone was wild-type in sequence. The β-glucuronidase gene (*gusA*) was transferred from pENTR-GUS (Invitrogen) into pSD-69 by LR recombination to give the control vector pSD-86. The cyan fluorescent protein gene (*CFP*) was cloned into pRRLhPGK.GFP.SIN18 from pECFP (Clontech, Saint-Germain-en-Laye, France) by standard techniques to give pSD-25. Lentivirus was produced by calcium phosphate transfection of 293T cells [16]. To transduce HMECs with multiple genes, infections were performed simultaneously with different viruses: cells were infected in suspension with the *ERα *and *BMI1 *vectors 24 hours after harvest from the patient (that is, after digestion with collagenase and dissociation of organoids to single cells), grown in suspension for 6 days in ultra-low-attachment dishes (Corning, New York, NY, USA), then dissociated, plated and infected with the *MYC*, *TERT *and *CFP *vectors. HMFs were infected with the CFP-expressing lentivirus in the initial experiment to facilitate identification in mice but were not transduced with *TERT *or transforming genes. The titre of each lentiviral batch was determined on primary HMECs. All infections were performed at a multiplicity of infection of 50 viral particles per cell.

### Antibodies

The following antibodies were used: antibodies against p14 (FL-132), p16 (M-156), MYC (9E10) (Santa Cruz Biotechnologies, Santa Cruz, USA); keratin 14 (RB-9020), keratin 18 (MS-142), progesterone receptor (PGR; Ab1), ERα (SP1), high molecular weight keratins (AB-3; Neomarkers, Stehelin, Basel, Switzerland); BMI1 (F6; Upstate, Lucerna-Chem AG, Lucerne, Switzerland); β-tubulin (Sigma); GFP (A11122) (Molecular Probes, Invitrogen); Ki67 (Novocastra, Newcastle, UK) and hTERT (R484) [17]. For Western blotting, goat anti-mouse or goat anti-rabbit antibodies coupled to horseradish peroxidase (Jackson ImmunoResearch, Newmarket, UK) were used, followed by chemiluminescent detection (Amersham, Little Chalfont, Bucks., UK). For immunofluorescent staining of tissue culture cells, samples were fixed with cold methanol. For staining of tissue, samples were fixed for 2 hours at 4°C in 4% paraformaldehyde and embedded in paraffin. Antigens were retrieved by boiling sections for 20 minutes in trisodium citrate buffer pH 6. Goat anti-mouse or goat anti-rabbit antibodies coupled to Alexa 488 or Alexa 568 (Jackson ImmunoResearch) were used for detection and the slides were mounted with 1,4-diazabicyclo [2.2.2]octane (DABCO; Sigma).

### Microarray analysis

RNA was extracted with an RNeasy kit (Qiagen, Hombrechtikon, Switzerland), amplified as described previously [18] and hybridised to U133Plus 2.0 gene chips (Affymetrix, CA, USA). CEL files were normalised with RMA [19]. The CEL files have been deposited in the GEO database under accession number GSE6548. DNA was extracted with a DNeasy kit (Qiagen), and 250 ng per chip was processed and hybridised to 50K *Hin*dIII SNP chips in accordance with the manufacturer's instructions (Affymetrix). The CEL files were analysed with CNAG 2.0 [20].

### Karyotyping

Cells were grown for 8 hours in HMM plus 100 ng/ml colcemid (Sigma). Metaphase spreads were prepared and stained with Giemsa (Sigma) essentially as described [21]. Fifty-five metaphase spreads of HMEC strains established from different donors were photographed and the chromosomes were counted.

### p53 assay

RNA was extracted from HMECs before injection into mice and from tumours in the fat pad with the use of an RNeasy kit (Qiagen). p53 status was determined by yeast functional assay with total RNA [22].

### Orthotopic xenograft

Animal experiments were authorised by the Veterinary Office of the Canton de Vaud, Switzerland. One million HMECs and 200,000 normal human mammary fibroblasts from separate cultures were mixed with 12.5% Matrigel (BD Biosciences) and injected into the fourth mammary gland of 8-week-old female NOD/SCID mice (NOD.CB17-*Prkdc*^*scid*^/J; Jackson Laboratory, Bar Harbor, ME, USA). Either the HMECs or the HMFs expressed CFP but never both: only one cell type in a single experiment was ever CFP-positive. The total time in tissue culture *ex vivo *before the epithelial cells were injected into the mammary fat pad was 28 days. Silicon pellets containing 1.5 mg of oestradiol [23] were inserted subcutaneously into the neck region of the experimental animals at the time that the cells were injected. For fulvestrant treatment, 5 mg of Faslodex (AstraZeneca AG, Zug, Switzerland) was injected subcutaneously at weekly intervals. The oestrogen pellets were not removed from fulvestrant-treated animals.

## Results

### BMI1 allows expansion of HMECs that express ERα

**Figure 1:**
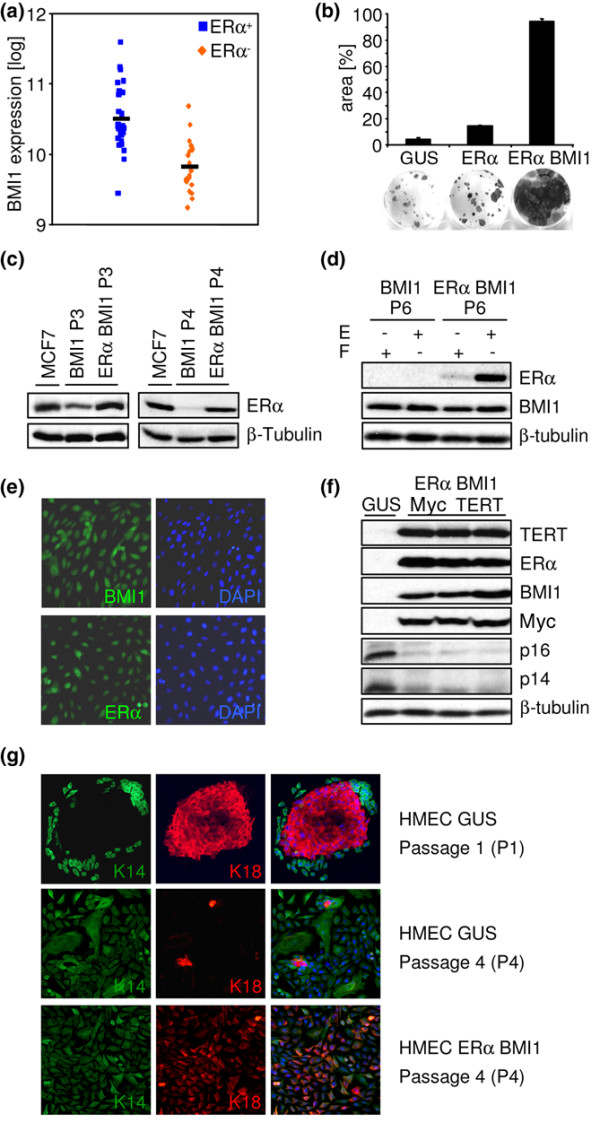
Expression of oestrogen receptor alpha (ERα) and BMI1 in human mammary epithelial cells. **(a) **Plot of microarray data [18] showing BMI1 expression in ERα-positive and ERα-negative breast tumours. BMI1 is significantly overexpressed in ERα-positive tumours (*p *< 0.001). **(b) **Colony formation assay. Human mammary epithelial cells (HMECs) transduced with either glucuronidase (*GUS*; a negative control gene) alone, *ERα *alone or *ERα *and *BMI1 *were fixed after growth for 10 days in the presence of oestrogen and then stained with crystal violet. The surface area covered with cells in the fixed plates was used to estimate growth. **(c) **Western blot for ERα and β-tubulin in MCF7 control cells or in passage 3 (P3, left) and passage 4 (P4, right) HMECs transduced with *BMI1 *alone or with *ERα *and *BMI1 *together. Endogenous ERα expression is progressively lost with passage. **(d) **Western blot for ERα, BMI1 and β-tubulin in passage 6 (P6) HMECs transduced with *BMI1 *alone or with *ERα *and *BMI1 *together. Before being harvested, cells were treated for 24 hours with 1 nM oestrogen (E) or 1 μM fulvestrant (F). Endogenous ERα expression is no longer detectable. Exogenous ERα is destabilised by fulvestrant. **(e) **Immunofluorescent staining of HMECs infected with *ERα *and *BMI1 *viruses for ERα (lower left panel) and BMI1 (upper left panel). 4',6-Diamidino-2-phenylindole (DAPI; right panels) was used to counterstain nuclei. ERα and BMI1 are both nuclear. **(f) **Western blot for TERT, ERα, BMI1, MYC, p14^ARF^, p16^CDKN2A ^and β-tubulin in HMECs infected with control virus (*GUS*, lane 1) or *ERα*, *BMI1*, *TERT *and *MYC *lentiviruses. HMECs from three different patients are shown in lanes 2 to 4. The transgenes are expressed, and BMI1 suppresses p14^ARF ^and p16^CDKN2A ^expression. **(g) **Immunofluorescent staining of HMECs for keratin 14 (K14, green) and keratin 18 (K18, red). DAPI (blue) was used to counterstain nuclei. At passage 1, HMECs infected with control virus and plated at clonal density formed mixed colonies with central K18-positive luminal cells and peripheral K14-positive myoepithelial cells (GUS P1). Passaging of these cultures led to progressive loss of luminal cells (GUS P4). HMECs infected with *ERα *and *BMI1 *viruses maintained K18 expression at passage 4, but individual cells were positive for both K14 and K18 (ERα BMI1 P4).

Microarray analysis of primary tumours [18] shows that the Polycomb-group gene *BMI1 *is overexpressed in ERα-positive tumours (Figure 1a; *r *= 0.62 for *ESR1 *versus *BMI1*, *p *= 4 × 10^-7^; similar results were obtained in several other microarray data sets) [24,25]. Because BMI1 suppresses growth arrest mediated by the Rb pathway [9] and the early growth arrest of HMECs has been linked to p16^CDKN2A ^expression [26], BMI1 expression is a potential escape mechanism for growth-arrested ERα-positive tumour cells. To test this, primary HMECs were transduced with lentiviral vectors expressing glucuronidase (GUS, which serves as a negative control), ERα and BMI1. Cells transduced with *GUS *or *ERα *alone formed small colonies. In contrast, transduction with *ERα *and *BMI1 *led to the formation of colonies that expanded to fill the dish in less than 10 days (Figure 1b). ERα expression from the lentiviral vector was stable with passage and comparable to that seen in the MCF7 breast cancer cell line (Figure 1c). In contrast, endogenous ERα expression was progressively lost with passage: it was still present at passage 3, substantially reduced at passage 4 (Figure 1c) and absent at passage 6 (Figure 1d). The exogenous ERα responded normally to regulation at the protein level, showing the expected decrease in level after treatment of the cells with the oestrogen antagonist fulvestrant (ICI 182,780/Faslodex; Figure 1d). Immunostaining confirmed the expression of both the *ERα *and *BMI1 *transgenes and showed that the encoded proteins localised to the nucleus, as expected (Figure 1e). To create an ERα-positive tumour model, cells expressing ERα and BMI1 were superinfected with lentiviruses expressing the c-*myc *oncogene (*MYC*) and the telomerase gene (*TERT/hTERT*). Western blotting was used to test for expression of the transgenes. *GUS*-transduced control HMECs did not express ERα, BMI1, MYC or TERT (Figure 1f, lane 1), whereas *ERα/BMI1/MYC/TERT*-transduced HMECs from three different donors showed robust expression of all four transgenes (Figure 1f, lanes 2 to 4).

**Figure 2:**
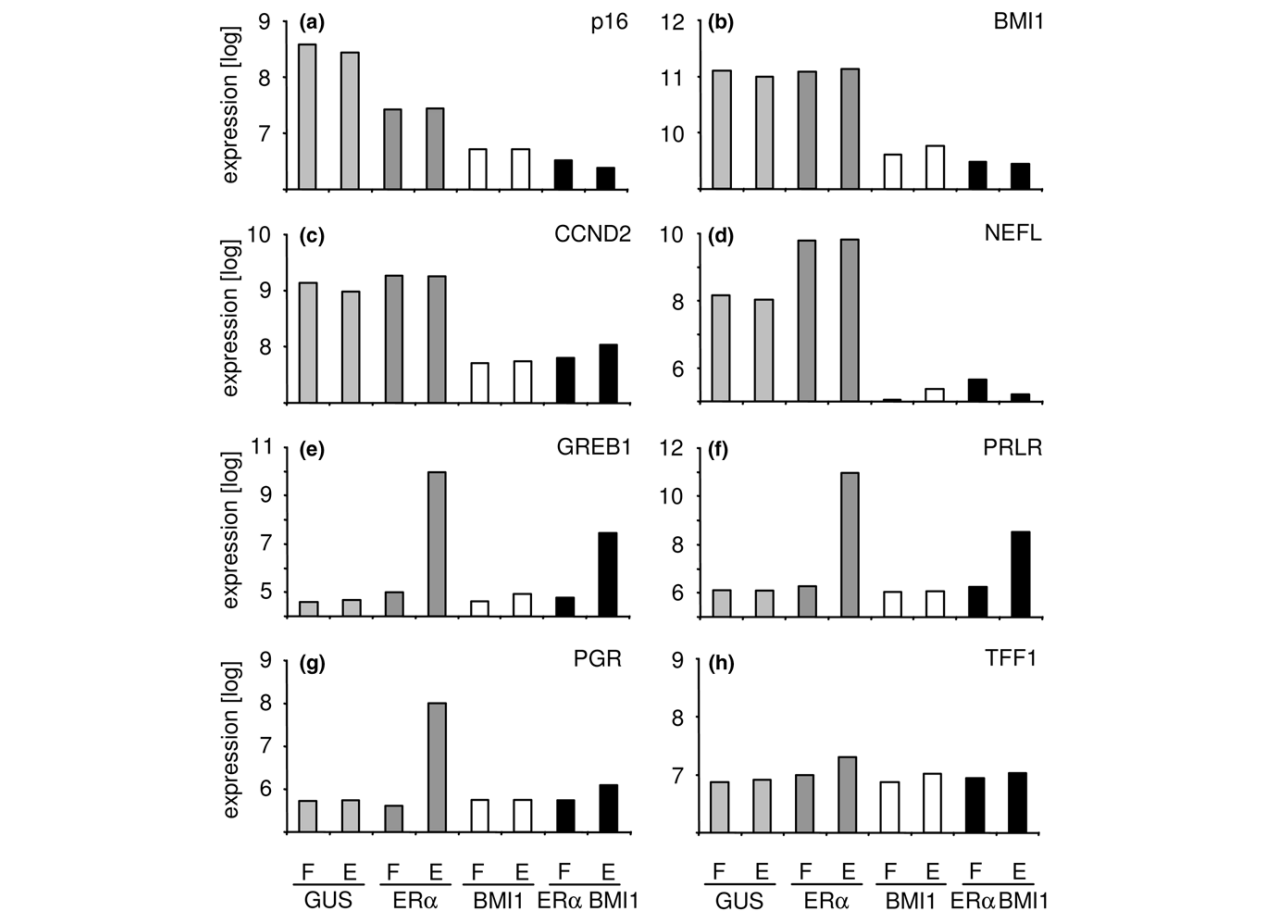
Gene expression profiles of HMECs transduced with *GUS*, *ERα *and *BMI1*. Cells transduced with *GUS *alone, *ERα *alone, *BMI1 *alone or *ERα *and *BMI1 *together were grown for 24 hours in the presence of oestrogen (E) or the oestrogen antagonist fulvestrant (F) to activate or block oestrogen signalling. Known BMI1 target genes (*p16*^*CDKN2A *^**(a)**, *CCND2 ***(c) **and *NEFL ***(d)**) are repressed by BMI1. Endogenous BMI1 is also repressed in *BMI1*-transduced cells **(b) **(the probe on the array detects only the endogenous transcript; the Western blots in Figure 1 show that overall BMI1 expression is increased in the *BMI1*-transduced cells despite the decrease in endogenous BMI1 expression). Many known oestrogen inducible genes are strongly induced by oestrogen receptor alpha (ERα) in the presence of oestrogen **(e) ***GREB1 *(gene regulated in breast cancer 1), **(f) **the prolactin receptor (*PRLR*) and **(g) **the progesterone receptor (*PGR*)), but some barely change (**(h) ***TFF1*). GUS, glucuronidase; HMEC, human mammary epithelial cell.

To test whether the *ERα *and *BMI1 *transgenes were biologically active, a microarray experiment was performed on cells after 10 days in culture. Oestradiol or fulvestrant was added to cells for 12 hours to activate or block ERα signalling, respectively. As expected, expression of the p16 gene (*CDKN2A*) was suppressed by BMI1 (Figure 2a). This was confirmed at the protein level by Western blotting for p14^ARF ^and p16^CDKN2A ^(Figure 1f; note that these cells were additionally transduced with *MYC *and *TERT*). Several genes recently shown by chromatin immunoprecipitation to be direct targets of Polycomb complexes were also repressed, including *BMI1*, *CCND2 *and *NEFL *(Figure 2b–d; the probe on the microarray detects only the endogenous *BMI1 *transcript) [27]. Expression of many ERα target genes, including *GREB1 *(gene regulated in breast cancer 1), *PGR *(the progesterone receptor gene) and *PRLR *(the prolactin receptor gene), was induced by oestradiol and blocked by fulvestrant, but only in cells transduced with *ERα *(Figure 2e–g). In contrast, several other classic ERα target genes, such as *TFF1*, showed little or no response to oestrogen (Figure 2h; the full data set has been deposited in the GEO database).

The medium used for both suspension and adherent culture was HMM [8]. Because it is based on neurosphere medium and the microarray showed the expression of some neural genes, such as *NEFL*, we sought to confirm the epithelial nature of the cells by staining for keratins. Control cells plated after one round of mammosphere culture formed three types of colony: pure keratin 18 (K18)-positive luminal colonies, pure keratin 14 (K14)-positive myoepithelial colonies, and mixed colonies containing both luminal and myoepithelial cells (Figure 1g, top panels). This is the same pattern as that reported by Dontu and colleagues [8] after mammosphere culture; similar observations have been made by other groups using different HMEC culture conditions [28-30]. At later passages, luminal cells were lost from the control cultures, resulting in the formation of increasingly pure K14-positive myoepithelial cell cultures (Figure 1g, middle panels). This is the expected result when HMECs are put into culture (reviewed in [31]). In contrast with the single-positive staining pattern of the controls, cells transduced with *ERα *and *BMI1 *were double-positive, staining for both K14 and K18 (Figure 1g, bottom panels). We conclude that the transduced cells are HMECs, the ERα and BMI1 proteins are correctly expressed and biologically active, and that BMI1 expression permits the outgrowth of ERα-positive colonies.

### Growth of ERα-transduced cells is dependent on oestrogen

**Figure 3:**
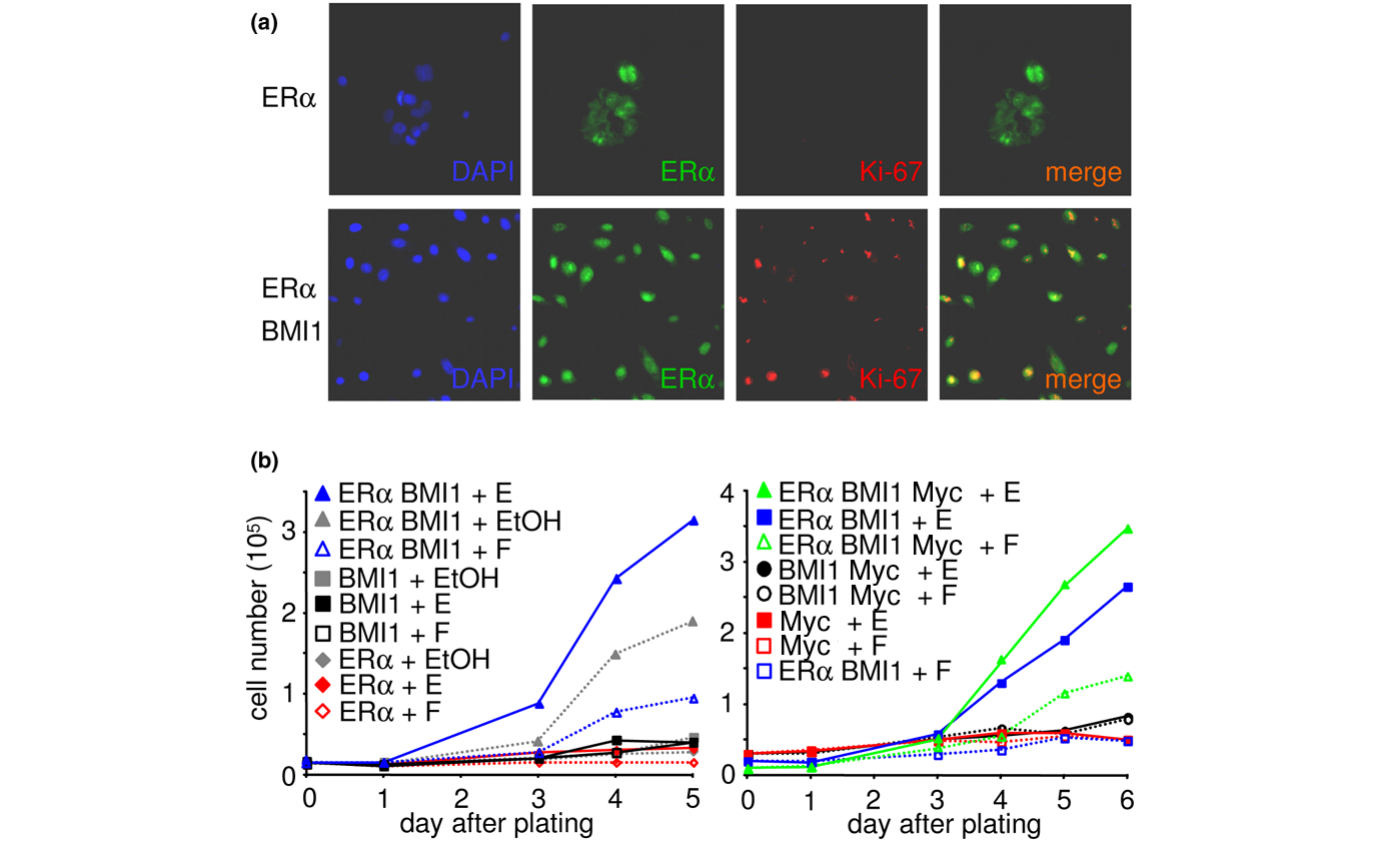
Growth of HMECs infected with the *ERα *virus is dependent on oestrogen. **(a) **Immunofluorescent staining for oestrogen receptor alpha (ERα) and Ki-67 of human mammary epithelial cells (HMECs) grown in the presence of oestrogen. HMECs infected with the *ERα *and *BMI1 *viruses together are Ki-67-positive, whereas HMECs infected with the *ERα *virus alone are Ki-67-negative. **(b) **Growth curves of HMECs expressing the indicated transgenes in the presence of oestradiol (E) or fulvestrant (F). The left and right panels show HMEC strains from two different patients. ERα and BMI1 are necessary for proliferation; transduction with *MYC *leads to a further increase in proliferation (right panel). Proliferation is increased by oestrogen and blocked by fulvestrant. The grey curves, labelled EtOH in the left panel, show the behaviour of cells in the absence of exogenous oestrogen or fulvestrant.

Oestrogen is a direct mitogen for ERα-positive tumour cell lines but not for normal mammary epithelial cells grown *in vitro*. The proliferative cells in the normal epithelium have been shown by immunostaining to be ERα-negative [5]. To test whether our virally transduced HMECs can proliferate in the presence of active oestrogenic signalling, oestradiol was added to the medium and cultures were stained for ERα and the proliferation marker Ki-67. Cells transduced with *ERα *alone were rarely Ki-67 positive (less than 1%), whereas a majority of cells transduced with both *ERα *and *BMI1 *were Ki-67 positive (more than 70%; Figure 3a). BMI1 thus overrides the negative control of proliferation by oestrogen in normal ERα-positive cells. To test whether oestrogen is a mitogen for the transduced cells, growth curves were performed in the presence of oestradiol or fulvestrant. Transduction with *BMI1 *alone can produce a proliferative burst in low-passage cells, but this disappears when endogenous ERα is lost (Figure 1d and data not shown). Passage 6 cells transduced with single genes failed to proliferate in any condition tested (Figure 3b). Cells expressing exogenous ERα and BMI1 were able to proliferate, and their growth was dependent on the presence of oestradiol in the medium (Figure 3b, solid blue curves) because it was blocked by fulvestrant (Figure 3b, dotted blue curves). Cells cultured in medium containing the drug vehicle (ethanol) without active drug had an intermediate growth rate (Figure 3b, left panel, grey curves); because this growth was blocked by fulvestrant it presumably reflects weak oestrogenic activity in the B27 supplement in the serum-free medium. Cells transduced with *MYC *alone failed to proliferate; cells expressing MYC, ERα and BMI1 grew slightly faster than *ERα/BMI1 *cells but were still responsive to oestrogen in the medium (Figure 3b, right panel, green curves). We conclude that forced expression of ERα and BMI1 bypasses normal controls on mammary epithelial cell proliferation and produces cells that are dependent on oestrogen for growth.

### Creation of an ERα-positive tumour model

**Figure 4:**
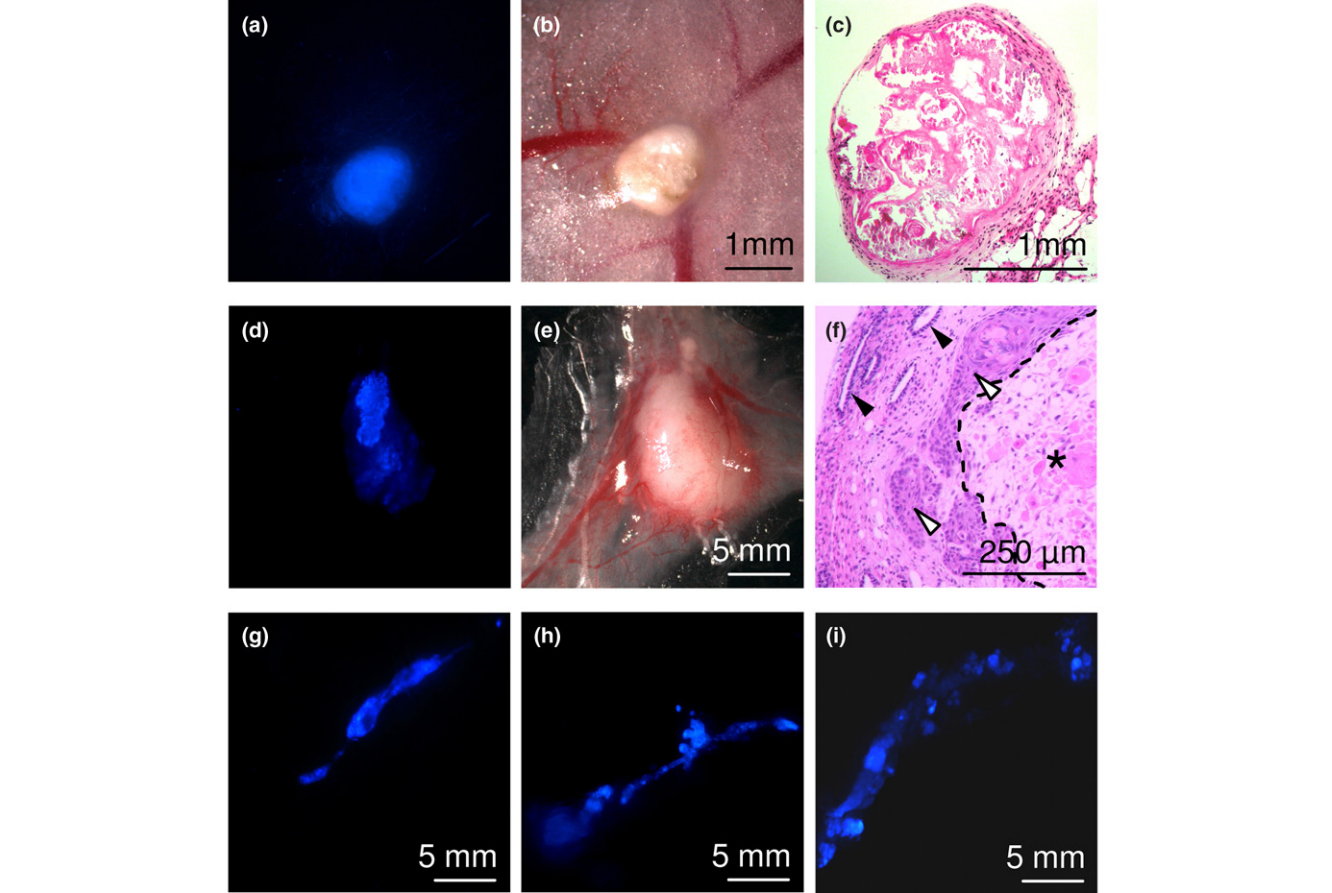
Tumour formation in the mouse mammary gland. One million human mammary epithelial cells (HMECs) expressing oestrogen receptor alpha (ERα), BMI1, TERT and MYC were injected into the inguinal mammary fat pads of NOD/SCID mice; 2 × 10^5 ^HMFs were injected simultaneously with the HMECs to promote engraftment. **(a-c) **In the absence of exogenous oestrogen, fibrotic nodules containing HMFs but lacking epithelial cells were all that remained after 60 days. In this experiment only the HMFs were labelled with cyan fluorescent protein (CFP). **(d-i) **In the presence of exogenous oestrogen, HMECs readily formed invasive tumours. In this experiment only the HMECs were labelled with CFP. Time after injection: (d-f) 5 days, (g) 14 days, (h) 21 days, (i) 35 days. (a,d,g-i), Fluorescence; (b,e), normal light; (c,f), haematoxylin/eosin staining. In (f) the open arrowhead indicates tumour, the filled arrowhead indicates mouse ducts, and the asterisk indicates necrotic cells at the site of injection. Note that HMFs were labelled with CFP in (a-c), whereas HMECs were labelled with CFP in (d-i): in each experiment only a single cell type was labelled with CFP.

To test whether the cells transduced with *ERα*, *BMI1*, *MYC *and *TERT *were transformed, they were injected into the inguinal mammary glands of 8-week-old female NOD/SCID mice. HMFs and Matrigel were injected with the epithelial cells to promote engraftment [32]. In the first experiment, the HMFs were tagged with a fluorescent marker protein (CFP). In the absence of exogenous oestrogen, compact CFP-positive nodules formed at the site of injection (Figure 4a,b). Histological examination showed that the nodules contained a capsule of fibrous tissue surrounding a core of necrotic epithelial cells (Figure 4c). Necrosis of the epithelial component was seen in 20 grafts of HMECs from three different patients in this experiment; this was confirmed in 12 grafts in a subsequent experiment in which the HMECs rather than the HMFs were tagged with CFP (Table 1, 'no treatment'). This shows that the *ERα/BMI1/MYC/TERT*-transduced HMECs are unable to survive in the normal hormonal milieu of adult mice.

**Table 1:** Tumour formation in NOD/SCID mice.

HMEC strain	No treatment	E2	E2 + fulvestrant
AJ	0/14	10/10	1/8
AK	0/14	12/12	3/6
U	0/4	11/12	0/8
Total	0/32	31/32	4/22

*ERα/BMI1/TERT/MYC*-transduced HMECs from three donors (AJ, AK and U) were injected into the inguinal mammary glands of NOD/SCID mice. Mice were divided into three groups: the control group (no treatment), which received no additional drugs (the cells were therefore exposed only to endogenous mouse oestrogen); the oestrogen-treated group, which received slow-release oestrogen pellets containing 1.5 mg of oestradiol (E2); and the fulvestrant-treated group, which received oestrogen pellets to allow engraftment followed after 32 days by weekly injections of fulvestrant to block oestrogen stimulation (E2 + fulvestrant). ER, oestrogen receptor; HMEC, human mammary epithelial cell.

The physiological level of oestradiol is higher in women than in mice, and it is frequently necessary to administer exogenous oestradiol to study human oestrogen-dependent phenotypes in mice [23]. The xenografts were therefore repeated in mice given slow-release oestradiol pellets. In these experiments, the HMECs were tagged with CFP. All except one of the 32 injected mammary glands developed tumours; the tumorigenicity of the transgene-expressing cells was confirmed by using HMECs from three different patients (Table 1, 'E2'). The process of engraftment and tumour formation was followed by killing mice at different time points (Figure 4d–f, day 5; then Figure 4g–i, days 14, 21 and 35, respectively). Five days after injection there was a large necrotic mass of tumour cells at the site of injection, accompanied by a vigorous vascular response (Figure 4e). On the surface of the mass, patches of brightly fluorescent CFP-positive epithelial cells were visible (Figure 4d). Histological examination confirmed that the main mass was necrotic (Figure 4f, the asterisked region bounded by the dotted line), presumably because it was insufficiently vascularised, but the brightly staining patches on the surface contained viable cells that were forming invasive tumour even at this early time point (Figure 4f, open arrowheads; closed arrowheads show mouse ducts). The absence of any lag suggests that the cells are quantitatively transformed. Every subsequent time point showed the presence of invasive tumour cells with a similar histological pattern: dense islands of squamous carcinoma adjacent to diffuse regions of invasive ductal carcinoma. We conclude that HMECs transduced with *ERα*, *BMI1*, *MYC *and *TERT *readily form oestrogen-dependent tumours in mice.

### ERα is active in the tumours

**Figure 5:**
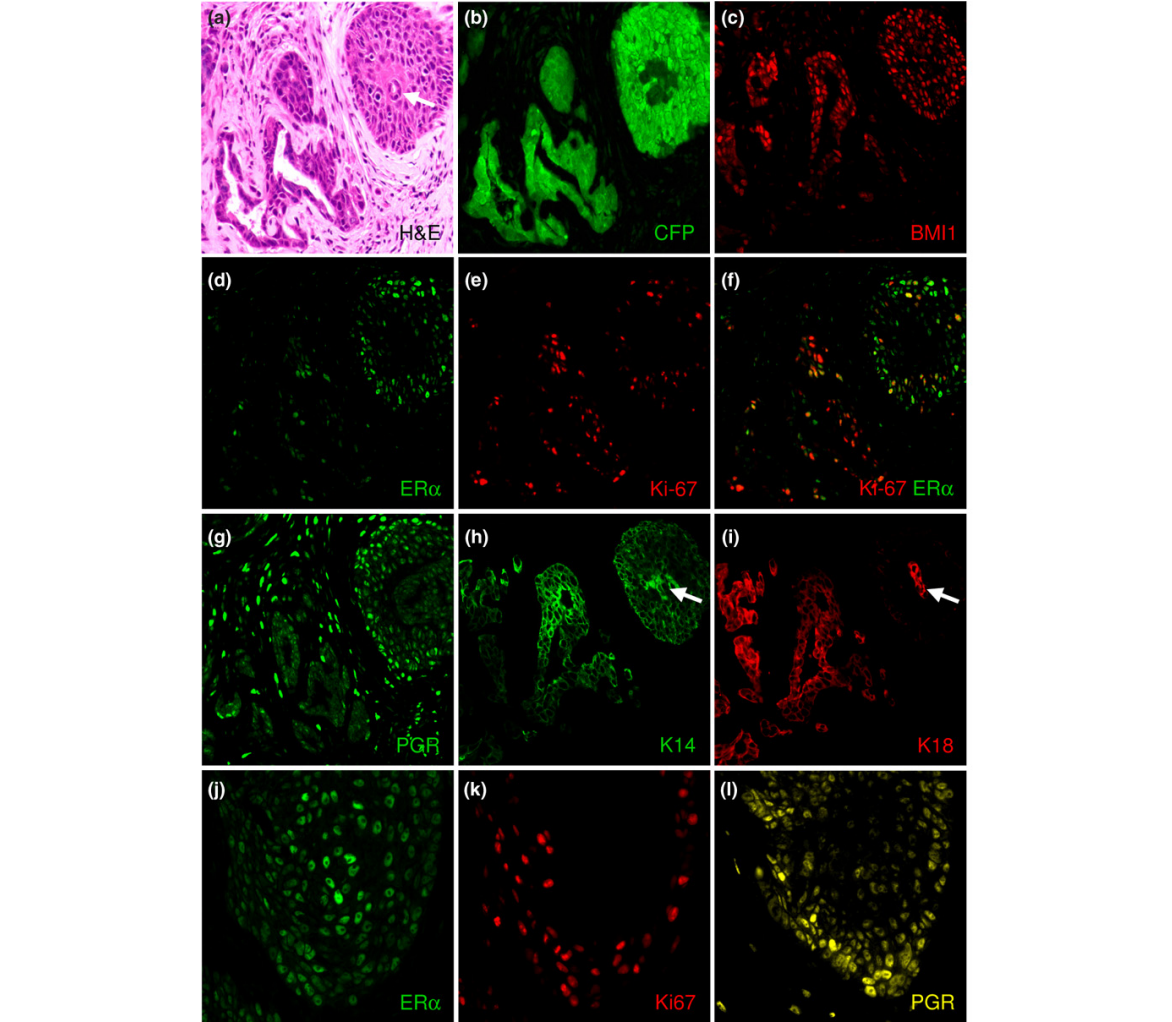
Immunofluorescent staining of tumours harvested 35 days after injection of *ERα/BMI1/TERT/MYC/CFP*-transduced HMECs. **(a-i) **Matched sections from one region of a tumour; **(j-l) **matched sections from a different region. The haematoxylin/eosin staining (H&E) in (a) shows the formation of tumours with dense squamous islands and diffuse infiltrating regions. The antibodies used for immunofluorescence in (b-l) are indicated in the lower right corner of each panel. The cyan fluorescent protein (CFP) staining in (b) and keratin 18 (K18) staining in (i) show that the tumour cells are derived from the injected human mammary epithelial cells (HMECs; the anti-K18 antibody is human-specific). BMI1 staining in (c) and oestrogen receptor alpha (ERα) staining in (d), (f) and (j) show that the HMECs retain nuclear expression of the transgenes. In (d-f), (j) and (k) it can be seen that some cells expressing ERα are also Ki-67-positive; (g) and (l) show expression of the *ERα *target gene progesterone receptor (PGR) in the tumour cells. There is a tendency, seen in (j) and (l), for cells with higher ERα expression to have lower PGR expression. In (h) and (i) it can be seen that the tumour cells are positive for keratins. The arrows in (a), (h) and (i) show a group of K18-positive glandular cells within a squamous island.

**Figure 6:**
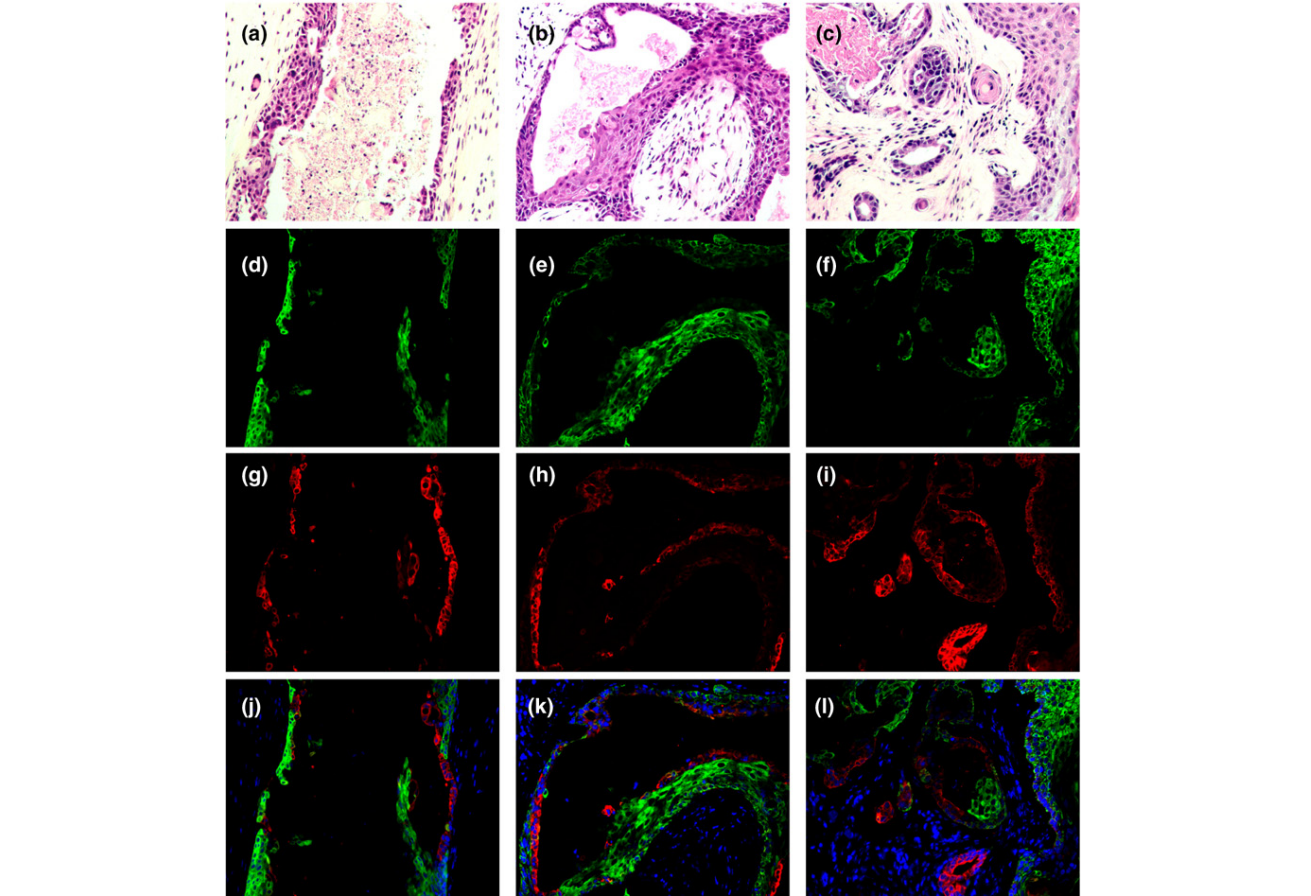
Keratin expression by tumours at different time points. The top panels show haematoxylin/eosin staining of tumours harvested at **(a) **14 days, **(b) **35 days and **(c) **60 days after injection of *oestrogen receptor alpha (ERα)/BMI1/TERT/MYC/cyan fluorescent protein (CFP)*-transduced human mammary epithelial cells (HMECs). The lower panels show matched sections immunostained for keratins. **(d-f)**, Keratin 14 (green); **(g-i)**, keratin 18 (red); **(j-l)**, merged image of the keratin 14 and keratin 18 signals together with a nuclear counterstain (4',6-diamidino-2-phenylindole (DAPI), blue).

Immunostaining of the tumours for CFP confirmed that all of the tumour cells were human in origin (Figure 5a,b). Matched sections were tested to verify that the *BMI1 *and *ERα *transgenes were expressed (Figure 5c,d,j). ERα staining was present in epithelial cells throughout the tumour (Figure 5d,f,j). PGR, which is a direct target of ERα, was also expressed throughout the tumour (Figure 5g,l). ERα staining was slightly weaker in regions of strong PGR staining (compare Figure 5j with Figure 5l). This is consistent with a previous report that ERα is degraded at the promoter of target genes being actively transcribed [33]. Staining for Ki-67 showed that the ERα-positive cells were actively dividing (Figure 5d–f,j,k). Ki-67 staining was stronger in invasive regions, as seen in the merged image in Figure 5f, where there is more Ki-67 staining in the invasive cells in the central and lower part of the figure, and more ERα staining in the squamous cells in the upper right part of the figure. Within squamous islands, Ki-67 staining was generally stronger in regions with weaker ERα staining (Figure 5j,k). The squamous islands were strongly positive for K14 (Figure 5h) and a group of keratins expressed in squamous epithelia (HMW keratin antibody against K1, K5, K10 and K14, data not shown). Invasive regions expressed both K14 and K18 (Figure 5h,i). Interestingly, the cells forming a glandular structure in the centre of the squamous island in Figure 5a (marked with an arrow) were positive for K18 despite being buried within the K14-positive squamous tissue. To explore further the keratin phenotype of the cells, tissue sections from tumours harvested at multiple time points were examined (Figure 6). In addition to regions double-positive for K14 and K18, the tumours contained single-positive regions expressing exclusively K14 or K18. The tube-like structures seen in Figure 4g–i were reminiscent of mammary ducts, with an outer layer of K14-positive cells and an inner layer of K18-positive cells (Figure 6d,g,j; 14 days). After 35 days the tubes had formed branches; most of the cells expressed K14 only but a significant proportion co-stained for K14 and K18, and some areas were strictly K18-positive (Figure 6e,h,k). After 60 days, most of the cells expressed only K14 and had formed squamous islands, but some K18-positive glandular structures were still present and double-positive cells were also seen, particularly in invasive zones (Figure 6f,i,l). We conclude that some of the tumour cells have the double-positive keratin phenotype of the ERα/BMI1 cells grown *in vitro *(Figure 1g), whereas others differentiate to single-positive cells that may represent a step of differentiation towards a luminal or myoepithelial fate, but with a strong tendency for the latter to progress to asquamous phenotype. ERα is expressed and transcriptionally active in the tumour cells, including cells that are actively proliferating.

### ERα-transduced cells are genetically stable

**Figure 7:**
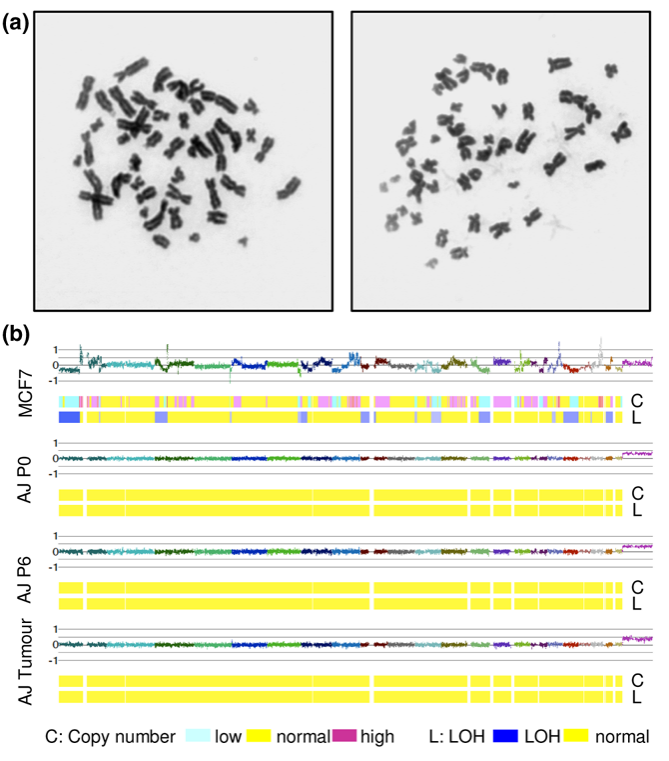
*ERα/BMI1/TERT/MYC*-transduced HMECs are genetically stable. **(a) **Representative karyotype of two different human mammary epithelial cell (HMEC) strains infected with *oestrogen receptor alpha (ERα)*, *BMI1*, *TERT *and *MYC *viruses after continuous in vitro passage for 60 doublings. **(b) **Copy number and loss of heterozygosity (LOH) plots. Each sample has three tracks: smoothed intensity, copy number prediction by Hidden Markov Model and LOH prediction. The horizontal axis shows each chromosome in turn, starting with the short arm of chromosome 1. In the intensity track each chromosome is given a different colour. The first set of tracks is from MCF7 cells, which were used as a positive control to demonstrate that the technique can detect amplification, deletion and LOH. The remaining three sets of tracks are, respectively, cells from reduction mammoplasty tissue of patient AJ at passage 0 (AJ P0), *ERα/BMI1/TERT/MYC*-transduced HMECs from the same patient just before they were injected into mice at passage 6 (AJ P6), and the same cells after recovery from established tumours (AJ Tumour).

Cells transformed with oncogenes *in vitro *invariably acquire additional genetic abnormalities, either during passage *in vitro *or subsequently as an adaptation to growth *in vivo*. Testing with a yeast functional assay that has been used extensively to test the p53 status of clinical samples [22] showed that p53 was wild type in the cell lines and tumours (data not shown). To test for other abnormalities, the karyotype was examined after 60 doublings *in vitro*. Cells from three different patients transduced with the *ERα*, *BMI1*, *MYC *and *TERT *viruses had a normal karyotype, with 46 chromosomes present in 54 out of 55 metaphases counted (Figure 7a). To screen for smaller genetic changes, the cells were tested on Affymetrix 50K SNP chips (Figure 7b). MCF7 cells were used a positive control because they contain multiple well-characterised amplicons and deletions. Cells were tested at three different time points, namely 6 days after removal from the patient, immediately before injection into the mouse, and after *in vitro *culture of cells recovered from established tumours. All of the samples had a normal genotype, with no evidence of amplification, deletion or loss of heterozygosity (Figure 7b). We conclude that the cells transformed with *ERα*, *BMI1*, *MYC *and *TERT *are genetically stable.

### Response to anti-oestrogen therapy

**Figure 8:**
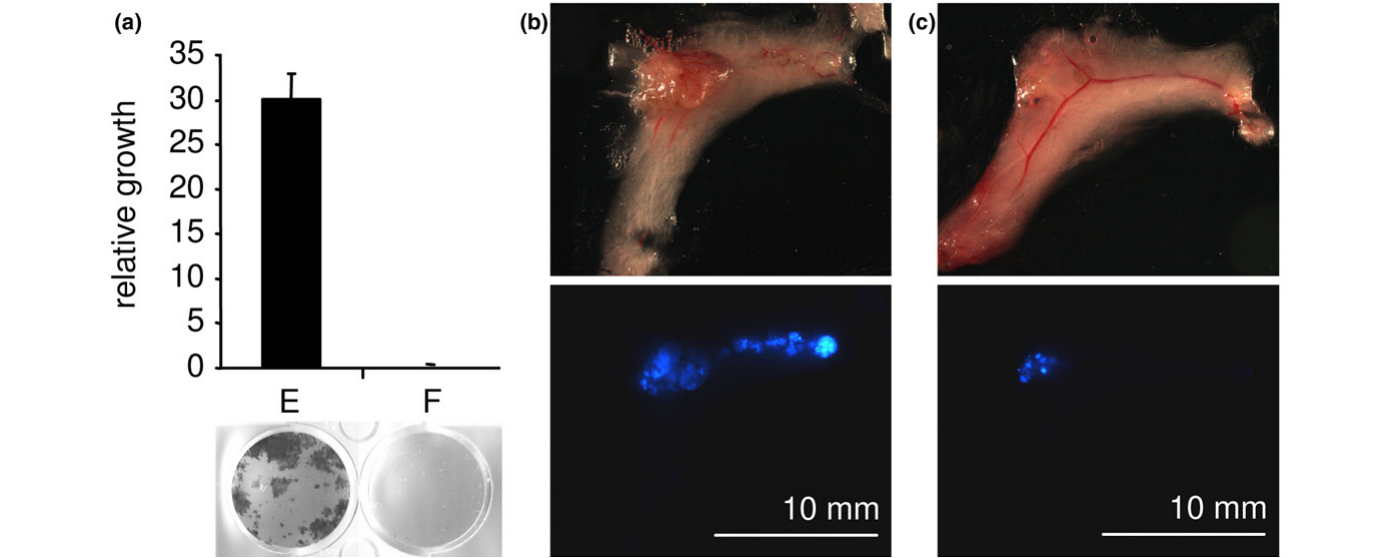
Response to anti-oestrogen therapy. **(a) **Tumour cells were recovered from a mouse, then grown in the presence of oestradiol (E) or fulvestrant (F). **(b,c) **Mammary glands of mice that received an oestrogen pellet before injection of the tumour cells, followed by either no additional treatment (b) or weekly subcutaneous injections of fulvestrant to block oestrogen signalling (c). Upper panels, normal light; lower panels, cyan fluorescent protein (CFP) fluorescence. Only the human mammary epithelial cells were infected with the CFP-expressing virus in this experiment.

To test whether the tumour cells retained their dependence on oestrogen signalling *in vitro*, cells explanted from primary tumours in mice were plated in medium containing either oestradiol or fulvestrant (Figure 8a). The cells showed strong inhibition of growth in fulvestrant, confirming that they remain oestrogen-dependent for growth *in vitro*. To test whether the cells were oestrogen-dependent *in vivo*, CFP-tagged *ERα/BMI1/MYC/TERT-*transduced HMECs from three different patients were injected into the inguinal mammary glands of NOD/SCID mice, and tumours were allowed to develop. Three groups of mice were tested. Two groups received an oestrogen pellet at the time of injection of the cells; one of these groups also received injections of fulvestrant starting 33 days after injection of the cells (Table 1, 'E2 + fulvestrant'). The third group was a control group that received an empty silicon pellet. Tumour response was scored by fluorescence microscopyfor CFP 40 days after the second group began treatment with fulvestrant (Figure 8b,c). In the group that received an oestrogen pellet alone, only 1 of 32 grafts had minimal or absent CFP fluorescence in the injected glands. In contrast, 18 of 22 grafts in the group that received fulvestrant displayed minimal or absent fluorescence (*p *< 10^-8^, Fisher's exact test). In the control group that received empty pellets, fluorescent epithelial cells were undetectable in any of the 12 injected glands, confirming the result of the experiment shown in Figure 4a–c. We conclude that the tumour cells remain responsive to anti-oestrogen therapy even after prolonged growth *in vivo*.

### Metastasis of tumour cells

**Figure 9:**
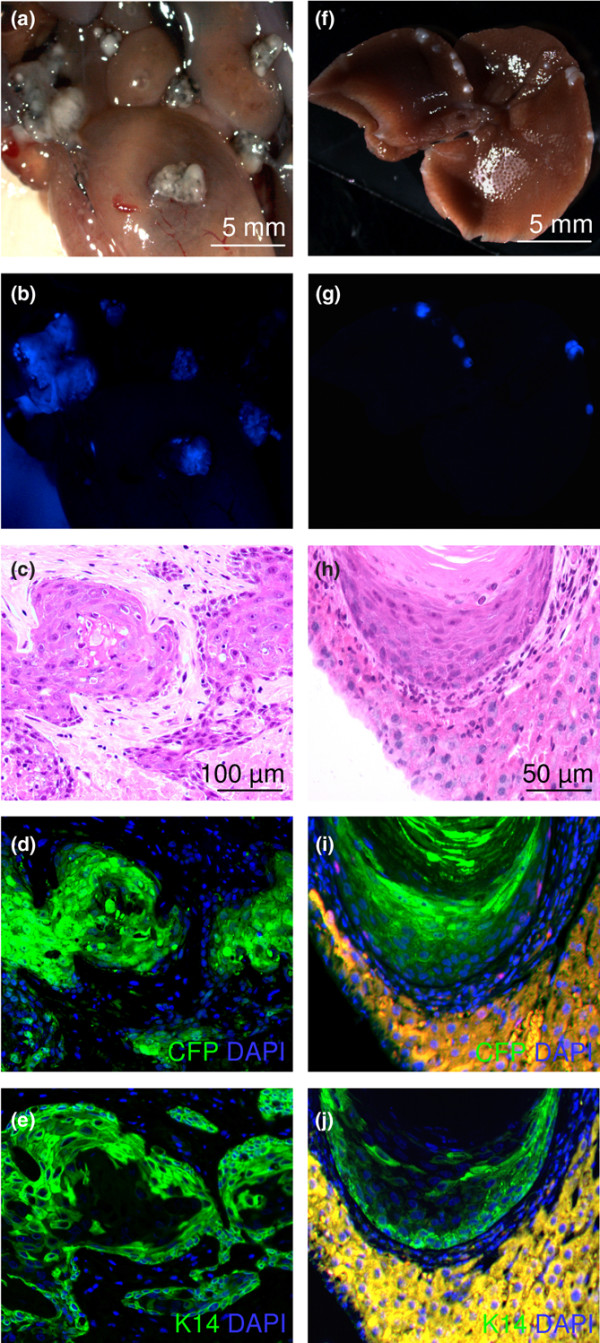
Metastasis formation after injection of transformed cells into the mammary gland. Metastases were observed from 30 to 90 days after injection of *oestrogen receptor alpha (ERα)/BMI1/TERT/MYC*-transformed human mammary epithelial cells (HMECs). **(a-e) **Peritoneal metastases 90 days after injection. **(f-j) **liver metastases 30 days after injection. **(a,f) **normal light, **(b,g) **cyan fluorescent protein (CFP) fluorescence, (c,h) haematoxylin/eosin staining, (d,i) CFP immunostain (green) with 4',6-diamidino-2-phenylindole (DAPI) counterstain (blue), (e,j) keratin 14 immunostain (green) with DAPI counterstain (blue). (c-e,h-j) Matched tissue sections of peritoneal and liver metastases, respectively. Liver tissue is stained yellow in (i) and (j) by non-specific binding of secondary antibodies coupled to Alexa 488 and Alexa 568. Only the HMECs were infected with the CFP-expressing virus in this experiment.

In 8 of 21 mice killed after 90 days there were metastases to multiple organs, including the liver and peritoneum (Figure 9; the HMECs were transduced with CFP). We also observed metastases in mice from the time-course experiment (Figure 4) as early as 30 days after injection of the cells. Metastases were present when the volume of the primary tumour was in the range 60 to 120 mm^3^. Thus, metastasis was not a late event occurring in the presence of an enormous tumour burden but an early event reflecting the intrinsic metastatic potential of the transformed cells. The histological appearance of the metastases was identical to that of the primary tumours (Figure 9). Immunostaining of the metastases for CFP confirmed that the metastatic cells were of human epithelial origin (Figure 9d,e). In contrast with the primary tumours, the metastases were positive for K14 but negative for K18 (Figure 9e,j). We conclude that transduction of normal HMECs with lentiviruses expressing ERα, BMI1, MYC and TERT confers the ability to form metastatic ERα-positive breast tumours.

## Discussion

We have developed a model for ERα-positive breast cancer by transformation of normal HMECs with *ERα*, *BMI1*, *MYC *and *TERT*. Metastasis occurred in 38% of the mice after 90 days. Previous attempts to make an ERα-positive model probably failed because ERα induces growth arrest and differentiation. We have not addressed the mechanism in this study, but transforming growth factor-β is known to restrain the proliferation of ERα-positive murine mammary epithelial cells [34]. Expression of BMI1 prevents differentiation and relieves the growth arrest, allowing the expansion of oestrogen-dependent HMECs in culture.

There are several differences between our culture system and those used previously. We grew the cells in floating mammosphere conditions before the first passage for several reasons. At a practical level, the final step after tissue digestion is a single-cell straining step; this facilitates efficient infection of the cells with lentiviral vectors less than 24 hours after the cells are removed from the patient. A second practical advantage is that fibroblasts do not survive in suspension, so floating mammosphere culture is an efficient way to eliminate fibroblasts. More importantly, it is based on techniques developed initially for propagation of neural stem cells [35] and later adapted for culture of HMECs [8]. The mammosphere approach enriches for bipotent progenitor cells that are capable of differentiating to myoepithelial and luminal cells, with production of milk proteins by the latter after treatment with prolactin in three-dimensional Matrigel culture [8]. The main difference between our approach and that of Dontu and colleagues [8] is that we used the same medium for suspension and adherent cell culture, and we omitted basic fibroblast growth factor. The medium is based on B27 [15], a serum-free medium supplement that is known to preserve the phenotype of human tumour cells in culture better than serum-containing media [36]. In our study, the relative importance of the medium versus the suspension culture is unclear but we have preliminary evidence that suspension culture may not be strictly necessary.

In the absence of a stem cell assay for HMECs it is not possible to state definitively whether mammospheres contain true human mammary epithelial stem cells (MaSCs). It is possible that the mammosphere approach enriches for mammary colony-forming cells (Ma-CFCs) rather than mammary repopulating units (MRUs) [37,38]. The nature of the cell initially infected with lentiviruses in our protocol is unknown because the infections were performed on the mixed population of cells present in reduction mammoplasty tissue. An intriguing question is whether cells expressing the recently identified murine MaSC markers would be more sensitive to transformation. Given the uncertainty surrounding the identity of human MaSCs, our main aim was to reduce the duration of growth *in vitro *to limit the potential for selection of adaptations to culture *in vitro*. The present study used 10^6 ^cells per fat pad injection, for which we needed to expand the cultures *in vitro *for a total of 28 days. We have preliminary results indicating that 5,000 cells are sufficient to form tumours, so it should be possible to greatly reduce the duration of culture *in vitro*.

Polycomb-group genes such as *BMI1*, *EZH2 *and *SUZ12 *have repeatedly been identified as adverse prognostic factors in breast cancer [11,12]. BMI1 is required for proliferation and renewal of stem cells in the brain and hematopoietic system. In mammospheres, *BMI1 *is thought to act as a point of convergence of the Wnt, Notch and Hedgehog signals that promote stem cell renewal [10]. BMI1 probably has at least a dual role, allowing cell proliferation by suppressing p14^ARF ^and p16^CDKN2A ^expression, and preventing differentiation through a more complex mechanism. Both processes are clearly visible in the microarray data reported here (Figure 2). ERα-positive tumours typically contain wild-type p53 and have fewer genomic changes than ERα-negative tumours [39]. The ERα-positive tumour model we have produced matches the human disease in this respect. The most likely explanation for the tumours to have retained wild-type p53 is that BMI1 suppresses p14^ARF ^expression [9]. Previous quantitative transformation models included genes such as those encoding simian virus 40 T antigen and p53^DD ^to inactivate p53 [3,4]. The MCF10A and MCF15 HMEC-derived cell lines show large differences in their DNA damage response despite both retaining wild-type p53 [40], and ERα-positive human breast tumours respond poorly to chemotherapy despite having wild-type p53. It is therefore important to note that although we have shown that the p53 cDNA is wild type, we have not shown that the p53 pathway is functional in our cells. The genes suppressed by BMI1 in ERα-expressing cells include many associated with neural and squamous differentiation. Suppression of these genes presumably favours proliferation by avoiding entry into a terminal differentiation program. We found that *BMI1 *itself was one of the genes suppressed by exogenous BMI1 expression. Bracken and colleagues showed by chromatin immunoprecipitation (ChIP) that the Polycomb-repressive complex 1 (PRC1) component CDX8 and the PRC2 component SUZ12 were present at the *BMI1 *promoter [27]. Suppression of PRC function by RNA-mediated interference (RNAi) led to derepression of genes with PRC proteins at the promoter [27]. On the basis of the ChIP data and the transcriptional response to RNAi against *BMI1*, *EED*, *SUZ12 *and *EZH2*, Bracken and colleagues suggested that PcG proteins autoregulate their own synthesis [27]. Autoregulation of BMI1 itself would by definition not have been detectable in their RNAi experiment, but when taken together with our results it is plausible that BMI1 suppresses its own expression through binding to its own promoter.

Wild-type *ERα *is not normally considered to be an oncogene, but behaves like one in our protocol. It is well known that ERα expression is rapidly lost from HMECs in culture. This is not solely a consequence of growth inhibition by ERα, because expression is still lost when cells are forced to express exogenous BMI1. In comparison with previous studies, the combination of genes we used to transform the cells seems rather gentle. In particular, we see no need to activate Ras signalling [2-4,41]. *TERT *was essential for successful transformation of HMECs in previous studies [3], but *BMI1 *and *MYC *can both activate TERT expression [42,43], so it is possible that *TERT *may not be required in our protocol. We included *MYC *in the protocol because *BMI1 *was originally identified as an oncogene that cooperates with *MYC *in lymphoma production in mice [13], *MYC *is commonly amplified in human breast cancer, and several groups have reported that *MYC *is required for HMEC transformation. Indeed, when Elenbaas and colleagues [3] used an HMEC transformation protocol lacking *MYC*, the cells spontaneously amplified *MYC *during culture *in vitro*. We used a wild-type *MYC (c-myc) *clone for our studies, rather than the activated form of *MYC *(T58A) used by Kendall and colleagues [4]. Despite the strong evidence that *MYC *is important, the requirements may differ when the selection conditions are changed, and we have preliminary evidence that *MYC *may not be required in *ERα/BMI/TERT*-transduced HMECs, at least for the initial stages of tumour formation.

It is intriguing that the transformed HMECs in our model can form polarised epithelial structures *in vivo *that express the correct luminal and basal keratins (Figure 6). This indicates that the double-positive keratin staining pattern *in vitro *is more likely to reflect a specific progenitor state than a loss of control of lineage-specific gene expression. Unlike the primary tumours in the mammary gland, the metastases were K18-negative. This suggests that the cells differentiate in response to signals from their local environment and that the mammary fat pad supplies specific signals that promote luminal keratin expression. Although the tumours contained regions of invasive adenocarcinoma, the predominant pathology was squamous carcinoma. Squamous differentiation is uncommon in human breast tumours. The squamous differentiation we observed in the NOD/SCID mice may reflect a general property of the mouse mammary fat pad model, a specific property of the target cell of the *in vitro *transformation protocol, or a defect in the transactivation of critical ERα target genes in the transformed cells. In comparison with the human breast, the mouse mammary gland contains much less fibrous connective tissue [44]. To promote engraftment of HMECs in the mouse mammary gland, human fibroblasts are commonly injected either at the same time as the HMECs or a few days earlier, to 'humanise' the stroma [32]. Human cancer-associated fibroblasts (CAFs) are similarly used to promote engraftment of human tumour cells in mice [45]. It is possible that the HMFs we injected together with the HMECs may have contributed to the squamous phenotype, but injection of our HMECs without HMFs led to the formation of tumours with similar kinetics and histology (data not shown). As mentioned above, we do not know the identity of the target cell of the transformation protocol. The mammosphere protocol enriches for mammary epithelial progenitor cells, but it is possible that expression of BMI1 promotes the survival of more differentiated cells or, conversely, forces progenitors to adopt a more stem-cell-like phenotype. HMEC protocols commonly give rise to squamous tumours in mice [3,46,47], so we consider it unlikely that the mammosphere protocol has led to the expansion of cells that are unrelated to mammary epithelium. Another possibility is that, despite expressing ERα, the cells are unable to respond appropriately to it. The gene expression profile of breast tumours is dominated by genes that are tightly associated with ERα status [48]. Many of these genes are direct targets of ERα but others are thought to represent markers of cell type. Some classic ERα target genes, such as the progesterone and prolactin receptor genes, were induced by oestradiol in the *ERα/BMI1 *HMECs, but others, such as *TFF1 *and *XBP1*, were not (Figure 2; the full data set is available in the GEO database under accession number GSE6548). The uninduced group includes many of the genes shown by RNA interference to require co-activation by FOXA1 [49]. Because FOXA1 is expressed only weakly in the transformed cells, transduction with a *FOXA1 *vector might lead to the activation of a broader range of ERα target genes and suppression of the squamous phenotype. More generally, there may be regulators of the differentiation programme of mammary epithelial cells, such as *GATA3 *or *TP73L*, that are not correctly expressed in the xenografts. We are currently testing these models to develop a transformation protocol that more faithfully reproduces the histology of human breast tumours.

It has long been known that ERα-positive breast tumours metastasise early, leading to distant relapse many years after excision of the primary tumour. In comparison with ERα-negative tumours, they have a better initial prognosis but this is followed by a relentless increase in breast cancer-specific mortality that continues even 15 years after treatment of the primary tumour [50]. It is tempting to speculate that the correct paradigm for these tumours is a low-grade lymphoma: a systemic disease characterised by few genetic changes and poor response to therapy. Intriguingly, there is a class of ERα-positive breast tumours that have only a single change on genomic profiling: an unbalanced translocation leading to gain of chromosome 1q and loss of chromosome 16q [51]. For this model to be correct, metastasis would have to occur early. Metastasis has not previously been reported in studies with HMECs transformed with defined oncogenes [3]. At early time points the transformed HMECs reported here invaded the fat pad rapidly, forming a prominent duct-like structure that arose either from cells deposited in the needle track or by the migration of cells out from the main mass (Figure 4g–i). We have not tested the invasive properties of the cells *in vitro*, but the specific combination of genes used to transform the cells certainly triggers an invasive and metastatic program *in vivo*. Because the key difference between this model and previous HMEC models is ERα expression, it is tempting to speculate that ERα itself has a critical role in the early metastasis of ERα-positive human breast tumours.

## Conclusion

We have created a new model for ERα-positive breast cancer by transduction of normal HMECs with lentiviruses expressing ERα, BMI1, MYC and TERT. The transformed cells are oestrogen-dependent for growth, wild-type for p53, diploid, and genetically normal as judged by hybridisation of tumour-cell DNA to SNP chips. The lack of secondary genetic changes and the high efficiency of tumour formation suggest that the cells are quantitatively transformed by the transgenes. The cells form disseminated peritoneal and liver metastases, a feature not previously seen with genetically defined, ERα-negative breast cancer models.

## Abbreviations

CFP = cyan fluorescent protein; DMEM = Dulbecco's modified Eagle's medium; ERα = oestrogen receptor alpha; GFP = green fluorescent protein; HMEC = human mammary epithelial cell; HMF = human mammary fibroblast; HMM = human mammosphere medium; K14 = keratin 14; K18 = keratin 18; MaSC = mammary epithelial stem cell; PGK = phosphoglycerate kinase; PGR = progesterone receptor; PRC = Polycomb-repressive complex; RNAi = RNA-mediated interference; SNP = single nucleotide polymorphism.

## Competing interests

The authors declare that they have no competing interests.

## Authors' contributions

SD carried out the lentiviral vector construction, tissue culture and mouse studies. ALN and SA carried out the microarray studies. MF participated in the pathological studies. SD, HB, CB and RI participated in the design and coordination of the study. SD and RI drafted the manuscript. All authors read and approved the final manuscript.
